# p53 reveals principles of chromatin remodeling and enhancer activation

**DOI:** 10.1093/nar/gkaf465

**Published:** 2025-06-06

**Authors:** Martin Fischer, Robert Schwarz, Konstantin Riege, Silke Förste, Katjana Schwab, Elina Wiechens, Alena van Bömmel, Steve Hoffmann

**Affiliations:** Computational Biology Group, Leibniz Institute on Aging—Fritz Lipmann Institute (FLI), Beutenbergstraße 11, 07745 Jena, Germany; Computational Biology Group, Leibniz Institute on Aging—Fritz Lipmann Institute (FLI), Beutenbergstraße 11, 07745 Jena, Germany; Computational Biology Group, Leibniz Institute on Aging—Fritz Lipmann Institute (FLI), Beutenbergstraße 11, 07745 Jena, Germany; Computational Biology Group, Leibniz Institute on Aging—Fritz Lipmann Institute (FLI), Beutenbergstraße 11, 07745 Jena, Germany; Computational Biology Group, Leibniz Institute on Aging—Fritz Lipmann Institute (FLI), Beutenbergstraße 11, 07745 Jena, Germany; Computational Biology Group, Leibniz Institute on Aging—Fritz Lipmann Institute (FLI), Beutenbergstraße 11, 07745 Jena, Germany; Computational Biology Group, Leibniz Institute on Aging—Fritz Lipmann Institute (FLI), Beutenbergstraße 11, 07745 Jena, Germany; Computational Biology Group, Leibniz Institute on Aging—Fritz Lipmann Institute (FLI), Beutenbergstraße 11, 07745 Jena, Germany

## Abstract

Pioneer transcription factors can bind to closed chromatin, initiating its opening and subsequent gene activation. However, the specific features that enable transcription factors to activate particular loci remain largely undefined. Here, we show that the transcription factor p53 unexpectedly initiates epigenetic remodeling at the majority of its binding sites and drives transcription at select loci. Our quantitative epigenetic data reveal that p53 establishes new enhancers, while quantitative transcription initiation analyses indicate that high local p53 abundance and sequence-specific binding are key features of sites where p53 successfully induces transcription. Surprisingly, we observed a spatial overlap between p53 binding sites and transcription initiation sites, suggesting a decoupling of these two events. Our results reveal that p53 activity unfolds across three distinct layers: histone modification, nucleosome eviction, and transcription initiation, with the latter driven by dynamic rather than static p53 DNA binding. These insights expand our understanding of the function of p53 by showing it not only actively initiates transcription but also broadly remodels chromatin. Overall, these findings offer a conceptual framework to explore how transcription factors regulate chromatin states and gene expression.

## Graphical Abstract

**Graphical Abstract:**
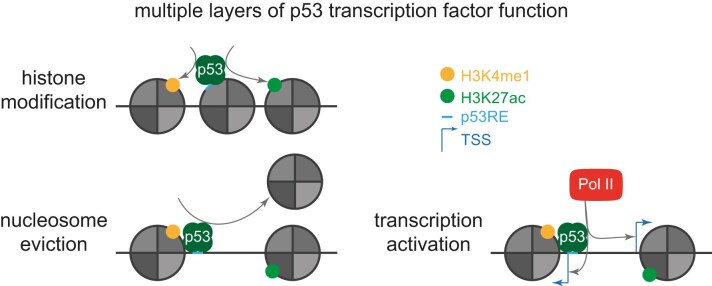

## Introduction

Transcription factors are central to genome regulation in development and disease by controlling enhancers and transcription activity [1–3]. However, it is largely unclear what distinguishes sites at which transcription factors exert regulatory effects from those sites at which they do not. In recent years, the well-known tumor suppressor p53 has emerged as a model transcription factor [4–7]. This is because p53 mediates particularly strong enhancer activity [8, 9] and has a well-understood gene regulatory network [10, 11], providing robust readouts. In addition, there is a large molecular toolbox to assess p53. For instance, small molecules such as Nutlin [12] allow a specific activation of p53, minimizing aberrant disturbances in other pathways.

While omics technologies have drastically expanded our understanding of how p53 functions as a transcription factor, several fundamental questions remain [13]. For instance, massively parallel reporter assays identified a globally strong enhancer activity driven by p53 response elements (p53REs) [6, 8, 9, 14–17]. However, in native chromatin, p53 activity appears to be much more restricted and context-specific [18], raising the question of what factors determine where p53 is active versus inactive. Another question is whether or not p53 functions as a *bona fide* pioneer factor. Unique to pioneer factors is their ability to bind sequence-specific sites in closed chromatin and initiate chromatin opening [3, 19]. Although p53 can access nucleosome-bound DNA [20–26], reports differ as to whether p53-mediated chromatin opening is rare [15, 22] or common [5, 27]. Recently, it has been reported that p53 binds preferentially to pre-established enhancers and has a limited capacity to remodel the local chromatin structure [28], suggesting that p53 may not have full pioneer factor capacities. In general, most p53 binding sites remain inaccessible and ineffective [22, 28], underscoring the need to understand the local contexts that enable its function. However, an integrated assessment of quantitative changes in chromatin characteristics and enhancer activity upon p53 induction is currently lacking.

Here, we quantitatively assessed DNA accessibility and nucleosome positioning by ATAC-seq, the enhancer histone modifications H3K4me1 and H3K27ac by CUT&Tag, and transcription start site (TSS) usage by CAGE-seq to systematically discern the regulatory impact of p53 at its binding sites in the human genome. Our data provide substantial evidence that p53 functions as a *bona fide* pioneer factor, including the establishment of *de novo* enhancers. Unexpectedly, our data reveal that p53 activity can be dissected into different layers and that p53 causes changes at the vast majority of its binding sites, with sequence specificity and high local p53 abundance being a reasonably good predictor of p53-dependent transcriptional activation. In addition, our data reveal an unexpected spatial overlap between p53 binding sites and TSSs, suggesting an uncoupling between DNA binding and transcription initiation by p53. Conceptually, our study provides a framework to better understand the local impact of transcription factors.

## Materials and methods

### Cell culture and drug treatment

MCF-7 (ATCC, Manassas, Virginia, USA) were grown in high-glucose Dulbecco’s modified Eagle’s media with pyruvate (Thermo Fisher Scientific, Darmstadt, Germany). Culture media were supplemented with 10% fetal bovine serum (FBS; Thermo Fisher Scientific), penicillin/streptomycin (Thermo Fisher Scientific), and nonessential amino acids (Thermo Fisher Scientific). Cells were tested twice a year for *Mycoplasma* contamination using the LookOut Detection Kit (Sigma–Aldrich, Darmstadt, Germany), and all tests were negative. Cell authentication was performed using morphological validation.

Cells were treated with Dimethylsulfoxid (DMSO) solvent control (0.15%; Carl Roth, Karlsruhe, Germany) or Nutlin-3a (10 μM; MedChemExpress, Monmouth Junction, NJ, USA) for 24 h.

### Transfection, RNA extraction, and reverse transcription semi-quantitative real-time PCR

Cells were seeded in six-well plates and reverse transfected with 10 nM Silencer Select siRNAs (#4390844 and #s607 from Thermo Fisher Scientific) using RNAiMAX (Thermo Fisher Scientific) and Opti-MEM (Thermo Fisher Scientific) following the manufacturer’s protocol.

Total cellular RNA was extracted using the innuPREP RNA Mini Kit (IST Innuscreen, Berlin, Germany) following the manufacturer’s protocol.

One-step reverse transcription and real-time polymerase chain reaction (PCR) were performed with a QuantStudio 5 using Power SYBR Green RNA-to-CT 1-Step Kit (Thermo Fisher Scientific) following the manufacturer’s protocol. We used *ACTR10* as a suitable control gene that we identified previously to be unaffected by p53 but expressed across 20 gene expression profiling datasets [29]. The following reverse transcription quantitative PCR (RT-qPCR) primers were used: *ACTR10* (forward: TCAGTTCCGGAAGGTGTCTT; reverse: GGACGCTCATTATTCCCATC), *TP53* (forward: CACATGACGGAGGTTGTGAG; reverse: ACACGCAAATTTCCTTCCAC), *CDKN1A* (forward: GGAAGACCATGTGGACCTGT; reverse: GGATTAGGGCTTCCTCTTGG), *ALDH3A1* (forward: GATGAGCCCGTGGAGAAGAC; reverse: GACAGGGGTCAGGTGCTTG), *HES2* (forward: CATCAACCAGAGCCTGAGC; reverse: GGACGTCTGCCTTCTCTAGC), *HMOX1* (forward: CCAGCAACAAAGTGCAAGAT; reverse: GTGTAAGGACCCATCGGAGA), and *NACC2* (forward: TGATGAACTGCCACCTGTGT; reverse: CAGCGTGTTCCTGTCAAAGA).

### ATAC-seq data processing

ATAC samples from biological quadruplets of MCF-7 cells, treated with Nutlin-3a and DMSO control, were obtained from GSE250017 and processed as described before [7]. That is, after initial data quality inspection using FastQC v0.11.9 (https://www.bioinformatics.babraham.ac.uk/projects/fastqc/), we applied Trimmomatic v0.39 [30] (5-nt sliding window approach, mean quality cutoff 22) for read quality trimming. Nextera transposase adapter sequence content was clipped using Cutadapt v2.10 [31]. Potential sequencing errors were detected and corrected using Rcorrector v1.0.4 [32]. Reads were aligned to hg38 using the mapping software segemehl [33, 34] v0.3.4 with adjusted accuracy (95%) and split-read option disabled. Non-mitochondrial alignments were selected and filtered for uniqueness and properly aligned mate pairs with Samtools v1.14 [35] as well as subsequently deduplicated by Picard *MarkDuplicates* v2.23.8 (https://broadinstitute.github.io/picard/). To account for Tn5 transposase cutting sites, 4 bp were clipped off from 5′-end of positive strand aligned reads, 5 bp otherwise, utilizing fgbio ClipBam v1.5.1 (https://fulcrumgenomics.github.io/fgbio). Peaks were then called from sub-nucleosomal fragments of size <120 bp using Macs2 v2.2.7.1 [36] (-f BED -q 0.01 --min-length 50 --max-gap 100 --call-summits --fe-cutoff 5 --nomodel --shift 75 --extsize 150) from BEDTools *bamtobed* v2.30.0 decoupled and re-formatted mappings [37].

We used DANPOS v3.0.0 [38] (forked from https://github.com/sklasfeld/DANPOS3) to obtain normalized read fractions from mononucleosome (180–240 bp) regions, as described previously [39].

### CUT&Tag and data processing

Biological duplicates of 100 000 MCF-7 cells treated with Nutlin-3a and DMSO control were used for CUTANA CUT&Tag assay (EpiCypher) according to the manufacturer's protocol version 1.5. Antibodies against H3K4me1 (#5326) and H3K27ac (#8173) were purchased from Cell Signaling Technology (Leiden, The Netherlands). The libraries were pooled and sequenced on a NextSeq 500 using a high-output kit v2.5 with 75 cycles (37 bp paired-end) run (Illumina).

Data processing was performed analogously as described earlier. Because of our high sequencing depth, we opted for deduplicating reads as suggested by the kit manufacturer. Aligned data were further filtered for fragments >120 bp and cleaned from reads overlapping ENCODE blacklisted regions (ENCFF356LFX) before being subjected to peak calling on sparse data using Macs2 without local lambda estimates (-f BED --nolambda -q 0.00001 --min-length 150 --max-gap 100 --broad --broad-cutoff 0.00001 --fe-cutoff 8 --nomodel --shift 0 --extsize 200). The “broad” peak option was selected to avoid any artificial fragmentation of peaks.

### ChIP-seq data processing

Single-end p53 ChIP-seq data were obtained from GSM2671290 and GSM2671296, which used DO-1 antibody that recognizes TAp53 isoforms [40] and processed as described earlier, while addressing Illumina universal adapter contaminants and not performing end-repair-clip. Recurrent p53 binding sites were taken from [41].

### CAGE-seq data processing

CAGE-seq data were obtained from GSE223512 and processed as described previously [7]. Briefly, Cutadapt was applied using the non-shifting 5′ adapter “XG” to clip a leading guanine and thus to correct for CAGE-seq’s typical 5′-end guanine addition bias before following initial preprocessing, including removal of Illumina universal adapter content, and alignment (see earlier). Unambiguously aligned reads were split into strand-specific subsets using Samtools to subsequently call strand-specific peaks using PEAKachu v0.2.0 (https://github.com/tbischler/PEAKachu) in adaptive mode, given all replicates. Peaks within a distance of 50 bp were merged with BEDTools *merge* [37]. CAGE-seq-detected TSSs (CTSSs) were obtained through BEDTools *genomecov* with the 5′-end coverage parameter, followed by BEDOPS v2.4.32 *max-element* [42] to determine TSS by local maxima.

### Differential expression analysis

Read quantification between Nutlin-3a and DMSO control samples was performed on merged peaks using featureCounts v2.0.3 [43], parametrized according to the CAGE-seq experiments library reverse strandness or non-strand-specific on all overlapping features for ATAC-seq and CUT&Tag, and subsequently tested for differential signals. Fold change and statistical significance were inferred using DESeq2 v1.34.0 [44], adjusted for multiple testing via the Benjamini–Hochberg procedure.

### Enrichment analysis of transcription factor binding

CistromeDB toolkit [45] was used to identify transcription factors that display ChIP-seq peak sets (top 1k peaks) that are significantly similar to the set of ATAC-seq or CAGE-seq peaks with significantly (False Discovery Rate (FDR) < 0.1) increased [log_2_fold change (FC) > 1] or decreased (log_2_FC < −1) signal.

### Chromatin states

Chromatin state information of untreated MCF-7 cells was obtained from EpiMap [46]. The 18 states were reduced to four states, namely promoter (TssA, TssBiv, TssFlnk, TssFlnkD, and TssFlnkU), enhancer (EnhA1, EnhA2, EnhBiv, EnhG1, EnhG2, and EnhWk), transcription (Tx and TxWk), and quiescent (Het, Quies, ReprPC, ReprPCWk, and ZNF/Rpts). Some regions were not annotated (n/a).

### Identification of putative target genes regulated through p53-bound enhancers

We incorporated enhancer-to-gene association data of MCF-7 cells from ENCODE (ENCFF617FJH) [47] to link CAGE-seq peaks (TSSs) to genes. To remove proximal promoter–gene associations and filter for distal enhancer–gene associations, we applied a distance threshold of 2.5 kb between CAGE-seq peaks and annotated target gene TSS. To identify target genes under potential control of p53-bound enhancers, we overlapped CAGE-seq peaks with p53 binding sites. We filtered for significantly and biologically meaningfully upregulated TSSs (CAGE-seq peak log_2_FC > 1, FDR < 0.1) and genes (gene log_2_FC > 0.5, FDR < 0.05).

### Positional enrichment of p53 around TSSs

We extended CTSSs by ±200 bp and overlapped them with p53REs to identify p53-specific TSSs. We used HOMER [48] with option *-size 200* to find the p53 motif enriched within the selected CTSS regions. The retrieved p53 motif was then applied to HOMER2 [6] with options *-size 401 -windows 19 -pkmer 3* to obtain a p53 position-specific enrichment within the CTSS regions.

### CTSS density around p53REs

The genomecov function of Bedtools v2.30.0 [37] was used to calculate the TSS coverage at each genomic position, considering only the 5′ position of the CAGE reads, so-called CAGE-seq-detected TSSs (CTSSs). We restricted our analysis to canonical p53REs containing two decameric half sites without a spacer (5388 out of 7705) [41] and extended these to 200 bp in the 5′ and 3′ directions. These regions were intersected with the genome-wide CTSS coverage and filtered for regions containing at least five CAGE-seq reads. The coverage for each position in a p53RE region was normalized by dividing the position-specific read count by the total read count of the corresponding p53RE region. Finally, the mean of the normalized read counts for each position across all p53RE regions was calculated and plotted for DMSO and Nutlin-3a data separated by EpiMap-derived chromatin states.

## Results

### p53 functions as a pioneer factor that increases DNA accessibility

**Figure 1.**
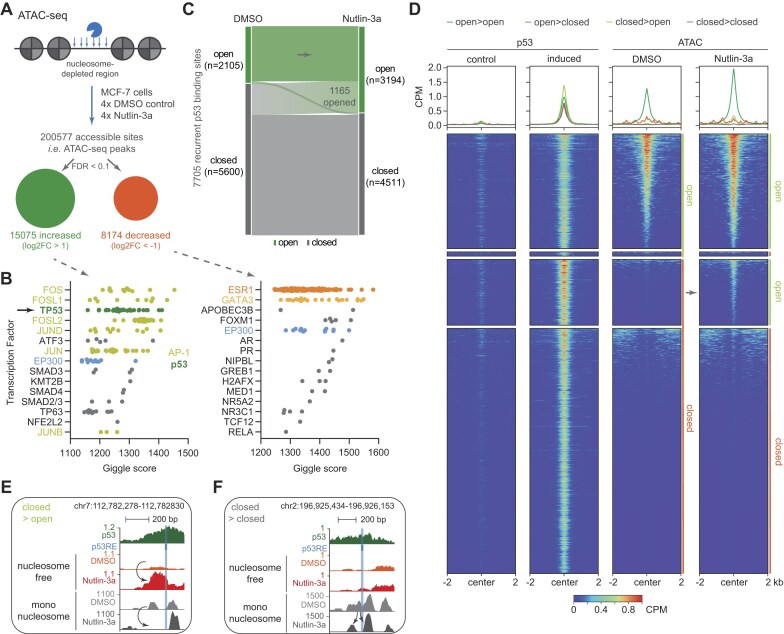
p53 is a pioneer factor that increases DNA accessibility. (**A**) ATAC-seq was performed on four biological replicates of Nutlin-3a and DMSO control-treated MCF-7 cells. Accessible sites were identified by peak calling, and their differential accessibility was assessed. (**B**) Enrichment of transcription factor binding sites that overlap sites with increased (left) or decreased (right) accessibility upon Nutlin-3a treatment. The top 15 transcription factors are displayed. (**C**) The presence (open) or absence (closed) of an ATAC-seq peak has been assessed at 7705 recurrent p53 binding sites, and changes between the Nutlin-3a and DMSO control conditions are displayed. (**D**) p53 ChIP-seq and ATAC-seq signals are displayed for the groups identified in panel (C). Regions sorted by ATAC-seq signal. Genome browser images displaying p53 ChIP-seq and ATAC-seq data at a p53 binding site (**E**) that became accessible and (**F**) that remained inaccessible upon Nutlin-3a treatment. The predicted p53RE is highlighted. p53 ChIP-seq and nucleosome-free ATAC-seq data were normalized to CPM. Mono-nucleosome ATAC-seq data were normalized by DANPOS according to library size and accessible DNA background.

To investigate differential DNA accessibility, we utilized Omni-ATAC-seq [49] upon p53 activation by the MDM2 inhibitor Nutlin-3a [12] in the widely used human breast cancer cell line MCF-7. We identified a total number of 200 577 accessible DNA regions, i.e. ATAC-seq peaks, in MCF-7 cells treated 24 h with Nutlin-3a and DMSO control. Among these regions, 15 075 and 8174 showed significantly increased and decreased accessibility, respectively (log_2_FC > 1/<−1 and FDR < 0.1) (Fig. 1A and Supplementary Table S1). When compared with publicly available transcription factor binding data, regions with increased accessibility showed a substantial overlap with binding sites of the transcription factors AP-1 (JUN/FOS) and p53 (Fig. 1B), suggesting that increased accessibility is strongly associated with p53 (TP53) binding. In contrast, regions with decreased accessibility showed a substantial overlap with binding sites of ESR1 and GATA3, which are known to cooperate at enhancers [50], and the latter of which is mutated in MCF-7 cells [51]. Both sets show substantial overlap with p300 (EP300) binding sites, a histone acetyltransferase that is involved in enhancer-mediated transcription [52], suggesting that many regions of differential accessibility represent enhancers.

DNA accessibility data at 7705 recurrent p53 binding sites [41] show that p53 binds to open chromatin at only about a quarter of sites (27%, 2105). Conversely, the majority of sites (73%, 5600) are in a closed chromatin state; i.e. no ATAC-seq peak was detected prior to p53 activation. Importantly, at 1165 of the closed sites, p53 activation by Nutlin-3a treatment leads to an opening of the previously inaccessible DNA (Fig. 1C). Given that our data were generated from bulk cells, simple peak-level data may not properly represent the DNA accessibility. Therefore, we also investigated quantitative changes in the ATAC-seq signal at p53 binding sites. Interestingly, activation of p53 by Nutlin-3a resulted in further increased accessibility at p53 binding sites already accessible prior to p53 activation. The small number of p53 binding sites that appeared to be closed after p53 activation have a comparatively high background signal and no apparent reduction in accessibility and thus appear to represent false-positive calls rather than a true decrease in accessibility (Fig. 1D).

In addition to assessing DNA accessibility, i.e. nucleosome-depleted regions derived from short ATAC-seq fragments, we used DANPOS [38] to infer single nucleosome positioning from the longer ATAC-seq fragments able to cover mono-nucleosome regions. Given that ChIP-seq-derived peak-level information on p53 binding is too imprecise to assess nucleosome positioning information, we used p53RE annotations, which were available for 6762 out of the 7705 p53 binding sites [41]. As expected, at constitutively open sites, we found that the p53REs were depleted of nucleosomes, while strong nucleosome signals were located next to them, with essentially no difference before and after p53 activation (Supplementary Fig. S1A). At loci where Nutlin-3a treatment made previously inaccessible DNA accessible, the data indicate a depletion of nucleosomes at p53REs upon p53 activation (Fig. 1E and Supplementary Fig. S1B and C), suggesting that p53 activity leads to nucleosome eviction, which is consistent with the increase in signals for accessible DNA. In addition, the data indicate that p53REs are preferentially located between nucleosomes before p53 binding (Supplementary Fig. S1B). Although ATAC-seq data are sparse at closed chromatin regions and should be interpreted with caution, our data indicate that p53 activation led to structured nucleosome repositioning at constitutively closed sites, with nucleosomes being shifted away from the p53REs (Fig. 1F and Supplementary Fig. S1D and E), while p53 activity did not lead to nucleosome eviction, as indicated by the absence of signals for accessible DNA. These data are in agreement with the ability of pioneer factors to induce nucleosome sliding [3] and suggest that p53 may be involved in chromatin remodeling at many more sites, albeit to a much lesser extent.

In contrast to p53, binding sites of the transcription factor RFX7, which is activated by p53 [29, 53], are largely accessible prior to p53 induction and do not show a significant increase in accessibility (Supplementary Fig. S2A). These data support a broad pioneer function of p53 and suggest that RFX7 has no pioneer activity.

### p53 establishes enhancers

**Figure 2.**
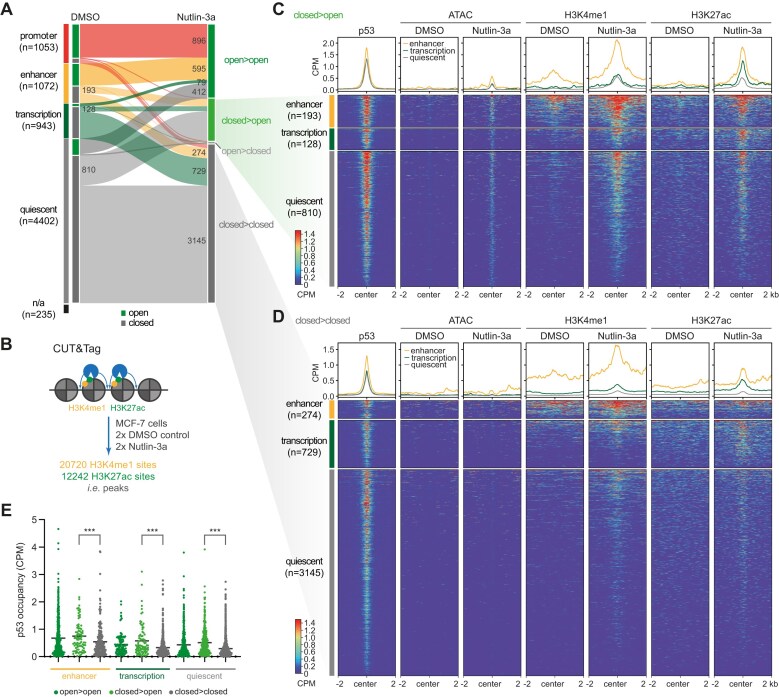
p53 establishes enhancers. (**A**) p53 binding sites were sorted by local chromatin state (promoter, enhancer, transcription, quiescent). In addition, they were sorted by the presence (open) or absence (closed) of an ATAC-seq peak in Nutlin-3a and DMSO control conditions. (**B**) CUT&Tag for H3K4me1 and H3K27ac was performed on two biological replicates of Nutlin-3a and DMSO control-treated MCF-7 cells. H3K4me1, H3K27ac, ATAC-seq, and p53 ChIP-seq signals are displayed for p53 binding sites that were closed in the DMSO control condition and open (**C**) or closed (**D**) in the Nutlin-3a treatment condition. Regions were sorted by average signal and p53 binding sites located in promoters were removed for visualization purposes because of the small group size and different signal scales. (**E**) p53 occupancy (CPM) at p53 binding sites in the different groups. Significance determined using a two-tailed Kruskal–Wallis test. ***P-value <.001.

It is well established that p53 successfully elicits transactivation at only a minority of its binding sites in the genome, while the precise context determining its activity is largely unknown [13]. To assess the importance of the preexisting chromatin state for p53 pioneering function, we integrated chromatin state information of MCF-7 cells from EpiMap [46] and inferred promoters, enhancers, transcribed regions, and quiescent chromatin. The majority (72%; 1519 out of 2105) of p53 binding sites with accessible DNA are located in promoters and enhancers already established before p53 activation. On the other hand, p53 binding sites where p53 activation made the DNA accessible were predominantly enriched in transcribed or quiescent chromatin (80%; 938 out of 1165) (Fig. 2A).

Given the increased DNA accessibility at p53 binding sites in quiescent chromatin, we asked whether p53 also leads to epigenetic remodeling at such sites. To this end, we used CUT&Tag [54] to assess changes in the histone marks H3K4me1, which is indicative of enhancers [55], and H3K27ac, which is thought to separate active from inactive enhancers [56] (Fig. 2B). Importantly, our data show that sites inaccessible prior to p53 activation showed little or no H3K27ac and H3K4me1 signals in the control condition. Upon p53 activation, H3K27ac and H3K4me1 signals were established at these sites (Fig. 2C), demonstrating that p53 can establish *de novo* enhancers. In addition, H3K4me1 and H3K27ac signals were also slightly increased at most sites that remained constitutively closed at the time of cell harvesting (Fig. 2D). Overall, our data show that Nutlin-3a treatment resulted in a substantial increase in H3K27ac and H3K4me1 signals at most p53 binding sites (Supplementary Fig. S3A). While p53 activation did not further increase the high H3K27ac levels at most constitutively open promoters, it substantially increased H3K4me1 and H3K27ac levels at constitutively open enhancers, other transcribed regions, and quiescent chromatin (Supplementary Fig. S3B). H3K4me1 and H3K27ac signals at sites putatively closed in response to the Nutlin-3a treatment showed no change or, if at all, an increase, further supporting that these represent false-positive calls (Supplementary Fig. S3C). Collectively, the data suggest that p53-mediated epigenetic remodeling precedes the opening of DNA. Notably, the p53 ChIP-seq signal was lower at p53 binding sites where p53 did not make DNA accessible compared with p53 binding sites where p53 succeeded in making DNA accessible (Fig. 2E). Presumably, higher local p53 abundance supports successful chromatin opening.

These results demonstrate that p53 can establish *de novo* enhancers. In addition, p53 also epigenetically alters sites that remain inaccessible.

### p53-induced transcription correlates with chromatin remodeling associated with high p53 abundance

**Figure 3.**
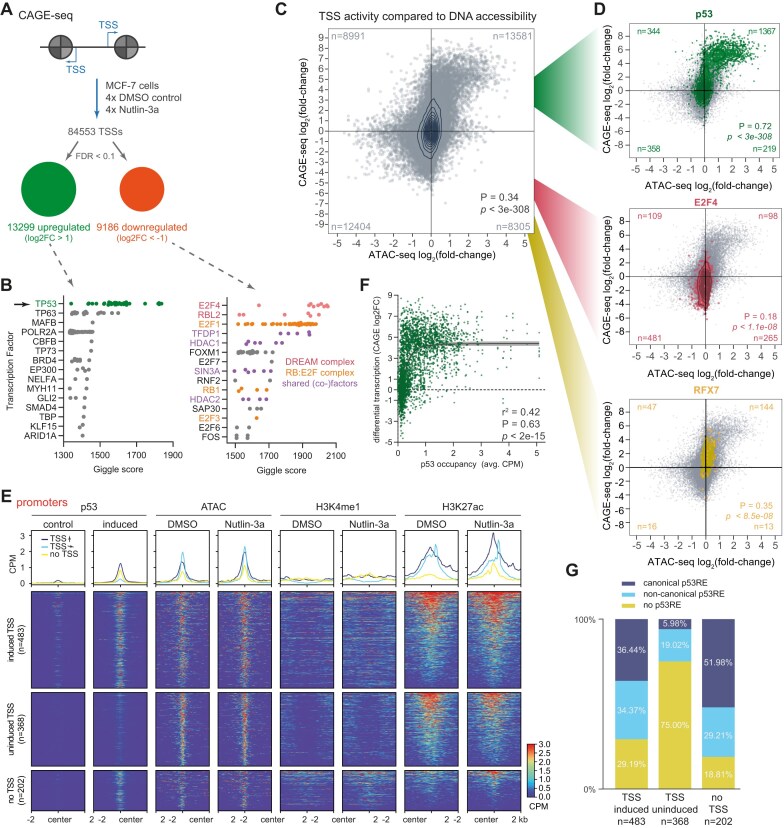
p53-induced transcription correlates with chromatin remodeling and requires high p53 abundance. (**A**) CAGE-seq was performed on four biological replicates of Nutlin-3a and DMSO control-treated MCF-7 cells. Significantly differentially regulated TSSs were identified. (**B**) Enrichment of transcription factor binding sites at sites with increased (left) or decreased (right) TSS activity upon Nutlin-3a treatment. The top 15 transcription factors are displayed. (**C**) Comparison of changes in TSS activity (CAGE-seq) and DNA accessibility (ATAC-seq) for all TSSs in accessible DNA regions (determined by ATAC-seq peaks) upon Nutlin-3a treatment. (**D**) Subsets of TSSs/accessible sites that overlap with p53 (top panel), E2F4 (middle panel), and RFX7 binding sites (bottom panel). (**E**) p53 ChIP-seq, ATAC-seq, H3K4me1, and H3K27ac signals at p53 binding sites located in promoter regions. Subgroups were determined by overlaps with an induced TSS (log_2_FC > 0.5), uninduced TSS (log_2_FC < 0.5), and no TSS. Regions sorted by H3K27ac signal. (**F**) Comparison of changes in local transcription (CAGE-seq) and p53 occupancy (CPM from ChIP-seq). Significance determined by two-tailed Spearman correlation. Sigmoidal fit obtained best *r*^2^. (**G**) The fraction of promoter p53 binding sites with a canonical p53RE, noncanonical p53RE, and no p53RE.

Notably, our finding that inaccessible p53 binding sites also showed considerable H3K27ac signals (Fig. 2D) is consistent with previous data showing that H3K27ac correlates with, but does not imply, enhancer activity [57]. Thus, although this mark is widely used, it may not be ideal for predicting productive p53 binding sites, i.e. sites at which p53 induces transcription. In particular, actual transcription has been shown to correlate strongly with enhancer activity [58–62]. To investigate the local transcriptional activity at p53 binding sites, we utilized CAGE-seq [63], a 5′-end RNA sequencing method that allows for the detection of TSSs and inferring the activity of promoters for coding and noncoding genes and enhancers [60]. We identified 84 553 TSSs, with 13 299 and 9186 TSSs displaying significant up- and downregulation with log_2_FC > 1 and <−1, respectively, and FDR < 0.1 (Fig. 3A and Supplementary Table S2). Notably, the differential TSS usage identified in MCF-7 cells upon p53 signaling showed a significant positive correlation with the differential TSS usage in U2OS and RPE-1 cells, and particularly for TSSs that overlap p53 binding sites (Supplementary Fig. S4A and B), suggesting that many sites are similarly regulated by p53 across cell lines. We extended the TSSs by 200 bp upstream for subsequent analyses. Compared with publicly available transcription factor binding data, TSSs with increased activity showed a substantial overlap with p53 binding sites (Fig. 3B), consistent with its function as transcriptional activator [64] and the increased accessibility at these sites (Fig. 1B and C). In contrast, TSSs with decreased activity showed a substantial overlap with binding sites of E2F4, p130 (RBL2), E2F1, DP1 (TFDP1), and RB (RB1) (Fig. 3B), which are critical components of the cell cycle transrepressor complexes DREAM and RB:E2F [65, 66]. These complexes repress cell cycle gene expression downstream of the p53–p21 signaling axis [10, 67–69]. Interestingly, E2F4 binding sites, which largely comprise binding sites of the DREAM complex [10, 65, 66], do not show a substantial difference in accessibility upon Nutlin-3a-induced p53 activation (Supplementary Fig. S2B). These findings are in support of a recently proposed model whereby the DREAM complex stabilizes +1 nucleosomes to repress transcription while binding to accessible promoter DNA [70]. Consistent with the recruitment of histone deacetylases to E2F4 targets [71, 72], H3K27ac levels were substantially reduced at these sites (Supplementary Fig. S2B).

Previously, transcription has been shown to be a robust predictor of enhancer activity [58, 62]. In general, changes in DNA accessibility showed a significant positive correlation with changes in TSS usage at these loci, but at the same time, many local changes in DNA accessibility and TSS usage are not associated with each other (Fig. 3C; Spearman correlation *ρ* = 0.34, *P* = < 3^−308^). At p53 binding sites, the correlation between changes in DNA accessibility and TSS usage was much stronger as compared to all loci (*ρ*_all_= 0.34 versus *ρ*_p53_= 0.72). In contrast, at E2F4 and RFX7 binding sites, the correlation, although significantly positive, was below (*ρ*_E2F4_= 0.18) and about the same correlation (*ρ*_RFX7_= 0.35), respectively (Fig. 3D), consistent with E2F4 and RFX7 binding only to accessible DNA (Supplementary Fig. S2). These data suggest a robust association between increased DNA accessibility and TSS induction at p53 binding sites.

Given that increased DNA accessibility and TSS induction were strongly, but not universally, associated with p53 binding sites, we asked which additional factors play a role in p53-induced transcription at a given site. Therefore, we categorized p53 binding sites based on TSS activity changes to investigate whether epigenetic marks predict local changes in transcription. Specifically, we separated p53 binding sites in promoters, enhancers, transcription, and quiescent chromatin by the presence of a CAGE-seq-detected TSS and whether it was induced (log_2_FC > 0.5) or uninduced (log_2_FC < 0.5) upon p53 activation. As expected, we detected a TSS near most promoter-associated p53 binding sites. In contrast, the minority of p53 binding sites in enhancers, transcription, or quiescent chromatin had a TSS. Intriguingly, about a third of p53 binding sites located in promoters showed uninduced TSSs (Fig. 3E). These uninduced promoter sites showed no increase in DNA accessibility, H3K4me1, or H3K27ac signals, whereas p53 binding sites that were associated with induced or no TSS showed a slight increase in these signals. In contrast, essentially no p53 binding site located in enhancers, transcription, or quiescent chromatin regions was associated with uninduced TSSs (Supplementary Fig. S5). We identified a strong positive correlation between p53 occupancy and transcription initiation locally, with a sigmoidal fit suggesting that a threshold of p53 occupancy may be required for transcription initiation at many loci (Fig. 3F). Notably, p53 binding sites related to uninduced TSSs show particularly weak p53 ChIP-seq signals (Fig. 3E), raising the question of whether or not these sites actually represent genuine p53 binding sites. Of note, low ChIP signal sites are frequently considered to be “sampling sites” rather than true binding sites [19, 73]. In addition, it is well known that the ChIP methodology has an open chromatin bias and can generate artifacts that present as false positive ChIP-seq signals [74, 75]. To this end, we assessed the presence of p53REs at these sites. We find that 70% and 80% of the p53 binding sites with induced and no TSS, respectively, contain a p53RE. In contrast, 75% of p53 binding sites with an uninduced TSS do not contain a p53RE (Fig. 3G), suggesting that these sites (*n* = 368 out of 7705; 4.8%) are mainly false positives. Moreover, these data suggest that a p53RE is a strong predictor of productive p53 binding sites at open chromatin. Previously, it has been suggested that only canonical p53REs consisting of two decameric half-sites without a spacer mediate productive p53 binding [14], but our data show that both canonical and noncanonical p53REs, i.e. three quarter-sites, 1 bp spacer separated half-sites, and single half-sites, are present at productive p53 binding sites (Fig. 3G), which is consistent with other studies that have identified noncanonical p53REs as functional [41, 76, 77].

Our data reveal that high local p53 abundance and the presence of a p53RE are recurrent and stable characteristics of epigenomically altered and productive p53 binding sites, i.e. sites where p53 induces chromatin remodeling and transcription. Together, our findings provide compelling evidence for the pioneer function of p53 and highlight a strong association between p53-induced DNA accessibility and transcription.

### p53-activated enhancers are associated with gene activation

Enhancers enable transcription factors, including p53 [78], to regulate gene expression across large genomic distances. Recent studies began incorporating chromatin interaction data to identify genes regulated by p53-bound enhancers genome-wide [28, 79, 80]. To uncover genes potentially regulated by p53 through enhancers, we utilized RNA-seq data from MCF-7 cells treated 24 h with Nutlin-3a and DMSO control. By integrating CAGE-seq and RNA-seq data, we observed a strong correlation between TSS activity and gene body expression (*ρ* = 0.76; Supplementary Fig. S6A). To establish enhancer TSS–gene associations, we incorporated chromatin interaction information of MCF-7 cells from ENCODE [47] and filtered interactions based on a minimum distance of 2.5 kb from the TSS—beyond which promoter-proximal regulation is expected to diminish [7, 81]. Our analysis revealed a statistically significant but modest correlation between differential TSS activity and the expression of distal genes engaging in chromatin interactions (*ρ*_all_= 0.21; Supplementary Fig. S6B). However, this correlation increased markedly when focusing on p53-bound TSSs (*ρ*_p53_= 0.31; Supplementary Fig. S6C), globally validating our CAGE-seq data and suggesting that p53 directly influences gene activation through enhancers. To identify genes regulated by p53-bound enhancers, we applied stringent selection criteria, filtering enhancer TSS–gene pairs in which the enhancer TSS showed significant activation (log_2_FC > 1 and FDR < 0.1) alongside a significant upregulation of the associated gene (log_2_FC > 0.5 and FDR < 0.05). This approach identified 146 candidate genes under p53 enhancer regulation (Supplementary Table S3), including well-characterized enhancer-driven p53 targets such as *CDKN1A* (p21) regulated through an enhancer located ∼9 kb upstream [82], *LINC00475* regulated through an intronic enhancer [83], and *GDF15* regulated through an enhancer located ∼10 kb upstream [16]. Additional genes likely upregulated through p53-bound enhancers include *ALDH3A1*, *HES2*, *HMOX1*, and *NACC2* (Supplementary Fig. S6D–H).

Collectively, these data underscore the widespread upregulation of genes through p53-bound enhancers, highlighting the critical role of enhancer-mediated regulation in the p53 transcriptional program.

### p53 preferentially directs transcription initiation to the p53RE and other sites within 100 bp

**Figure 4.**
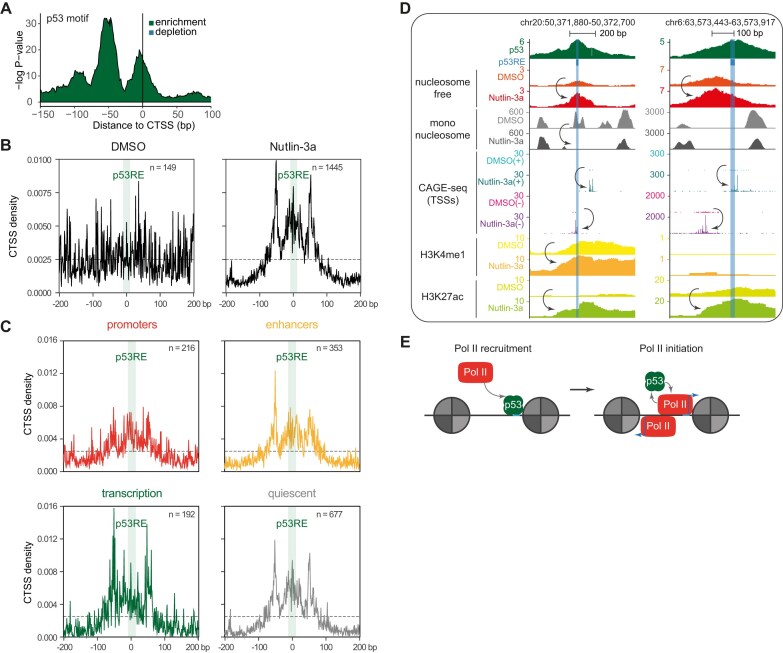
p53 preferentially directs transcription initiation to the p53RE and other sites within 100 bp. (**A**) Positional p53 motif enrichment relative to the CAGE-seq-detected TSS (CTSS) identified by HOMER2. The density of CTSSs at ±200 bp around canonical p53REs of p53 binding sites in (**B**) Nutlin-3a and DMSO control samples and (**C**) the Nutlin-3a data separated by EpiMap-derived chromatin states. (**D**) Genome browser images displaying CAGE-seq data among p53 ChIP-seq, ATAC-seq, and CUT&Tag (H3K4me1 and H3K27ac) data at two p53 binding sites. The predicted p53RE is highlighted. p53 ChIP-seq, nucleosome-free ATAC-seq, and CUT&Tag data were normalized to CPM. Mono-nucleosome ATAC-seq data were normalized by DANPOS. CTSSs were coverage normalized. (**E**) Dynamic p53 binding is required for Pol II initiation after its recruitment to the edges of nucleosome-depleted regions.

With precise quantitative TSS data at hand, we asked whether there is a preferred distance at which p53 directs Pol II to initiate transcription. It has been reported that p53 binding sites lack preferential spacing toward TSSs [6]. However, the results were based on massively parallel reporter assays lacking the native chromatin context. Using our quantitative TSS data from native chromatin, we find that p53 motifs are preferentially located at the TSS and within 100 bp upstream (Fig. 4A). To obtain more detailed spatial information, we used precise p53RE annotations, which are available for most p53 binding sites [41]. We extended the p53REs by 200 bp upstream and downstream from the center and assessed the relative frequency of TSS usage at these 400 bp intervals. In DMSO control cells, only 115 sites showed TSS activity, which was rather randomly distributed across the 400 bp interval. However, CAGE-seq-detected TSSs were observed at 1445 sites in Nutlin-3a-treated cells. Surprisingly, TSS usage was highly enriched at two distances, one showing a peak at ∼50 bp from the p53RE and the other showing a peak precisely at the p53RE (Fig. 4B). Separation of the sites based on the chromatin state prior to p53 activation showed that the distance of TSSs to p53REs was more uniform at promoters. At the same time, it was more constrained to the preferred distances of ∼0 and ∼50 bp at enhancers, transcribed DNA, and quiescent chromatin (Fig. 4C). The more uniform TSS distribution at promoters may be affected by the presence of other transcription factors. We speculated that fewer transcription factors might bind to loci inaccessible before p53 binding, and thus, we separated the p53 binding sites by DNA accessibility, i.e. overlap with an ATAC-seq peak. However, the distance of TSSs elicited at p53 binding sites was essentially the same at sites that were open and closed prior to p53 activation (Supplementary Fig. S7), suggesting that p53 retains its preference and other nearby transcription factors may affect TSS location at only a minority of loci. Interestingly, the preferred distance of TSS initiation is within the length of DNA wrapped around a single nucleosome. This suggests that p53 may preferentially direct transcription initiation to the edges of a nucleosome-depleted region from a p53RE located at one of the edges. When we inspected individual sites, we observed cases where the p53RE marked one edge of the nucleosome-depleted region while p53 directed transcription initiation to both edges (Fig. 4D).

Taken together, these results show that p53 directs Pol II to initiate transcription at the p53RE and within 100 bp, possibly representing edges of DNA stretches that can wrap around one nucleosome (Fig. 4E).

## Discussion

**Figure 5.**
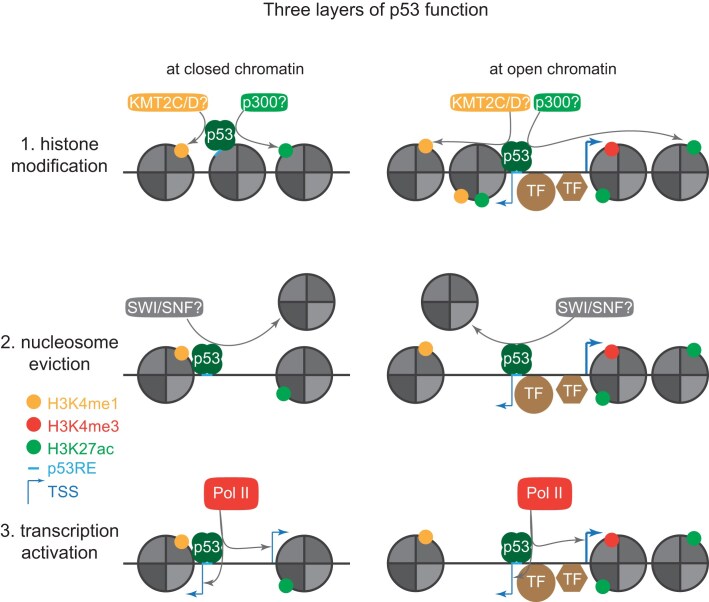
Model of p53 function at the DNA. Three layers of p53 function: histone modification, nucleosome eviction, and transcription activation.

Our study provides quantitative data demonstrating a broad pioneering function of p53 that includes opening chromatin that was inaccessible before p53 activation and further increasing the accessibility at loci that were already accessible (Fig. 1D and Supplementary Fig. S3B). In addition, our data demonstrate that the increase in DNA accessibility and the induction of transcription by p53 are closely linked (Fig. 3D). Furthermore, our data provide compelling evidence that p53 can establish *de novo* enhancers (Fig. 2C), in contrast to a recent study suggesting that p53 function is restricted mainly to pre-established enhancers [28]. Collectively, our data indicate that p53 exerts three distinct functions at chromatin loci (Fig. 5). First, p53 mediates a local epigenetic remodeling of chromatin to establish enhancer marks in the absence of a promoter or enhancer and further strengthens the epigenetic structure at already established enhancers (Fig. 2C and Supplementary Fig. S3B). It seems likely that p53 achieves this through its reported ability to recruit the histone methyltransferases KMT2C (MLL3) and KMT2D (MLL4) [84], which deploy H3K4me1, and the histone acetyltransferase p300 [85, 86], which deploys H3K27ac. Indeed, p300 is essential for enhancer activation by p53 [4, 85]. Second, p53 attempts to make inaccessible DNA accessible and to further increase the accessibility at open sites (Fig. 1D and Supplementary Fig. S3B). It seems likely that this is accomplished by the reported ability of p53 to recruit the SWI/SNF chromatin remodeling complex [87]. Third, p53 attempts to induce transcription (Fig. 3E and Supplementary Fig. S5).

One of the long-standing questions is what distinguishes sites at which p53 can induce transcription from sites at which it cannot [13]. First of all, our data suggest that p53 function can be separated into at least three layers, namely histone modification, nucleosome eviction, and actual transactivation (Fig. 5). When all of these functional layers are considered, our data unexpectedly suggest that p53 causes changes at the vast majority of its binding sites (Fig. 1F and Supplementary Figs S1D and E and S3A). However, each functional layer seems to be a prerequisite for the next. Indeed, H3K4 mono-methylation is known to occur before nucleosome eviction [19], and nucleosome-depleted DNA is a well-established prerequisite for the induction of transcription [1]. Although nucleosome depletion is also thought to be a prerequisite for H3K27ac [19], our data show that p53 can induce H3K27ac also at loci where chromatin remains closed (Fig. 2D). Importantly, p53 seems unable to exert its full productivity, i.e. transactivation, at most chromatin loci, which is consistent with the prevailing models of p53-mediated transcription that find p53 to function only at a minority of its binding sites [18]. Our data suggest that p53 ChIP-seq signal intensity, which is indicative of local p53 abundance, is a reasonably good predictor of productive p53 binding in general. That is, while at closed chromatin a rather low p53 abundance is restricted to mediate histone modifications (Fig. 2D), a higher p53 abundance is required to make DNA accessible (Fig. 2E) and to induce transcription (Fig. 3F). Based on these data, we propose a model of p53 productivity that is driven by its local abundance, which must overcome a local threshold. Indeed, threshold-based models of the p53 function are increasingly appreciated [88]. Although this rather simple model clearly does not include all the contexts that affect p53 function and thus does not account for all chromatin loci, e.g. sites at which local cofactors such as TRIM24 [5] affect p53 function, our data suggest that it accounts for a surprisingly large number of loci.

It is well known that transcription initiation typically occurs at the edges of nucleosome-depleted stretches of DNA and that these edge regions represent so-called core promoters at which the pre-initiation complex can be assembled [1, 2]. Recently, results from massively parallel reporter assays suggested that p53 binding lacks a preferential spacing toward TSSs and may activate transcription regardless of its position relative to the TSS [6]. In contrast, our data consider the native chromatin context and reveal clear preferences in the spacing between p53RE and TSSs. Our data demonstrate that p53 directs transcription initiation, at least in part, to the p53RE (Fig. 4A–D), suggesting that p53REs can act as core-like promoter motifs. In fact, this feature may be due to the CA, CG, and TG dinucleotides that are central to the initiator motif at which Pol II initiates transcription [89, 90] and that occur in the p53RE core. Notably, most models of transcription factor-mediated transcription initiation postulate that transcription initiation occurs at the time when transcription factors are bound to the nucleosome-depleted DNA and that this process is interrupted as soon as or shortly after activator transcription factors dissociate from the DNA [1, 2, 91]. In the case of p53, there is also a close temporal relationship between DNA occupancy and induction of transcription [40, 92]. Our data reveal that p53 unexpectedly directs transcription initiation to p53REs (Fig. 4A–D), suggesting a decoupling of p53 binding and transcription initiation because p53 binding to the p53RE blocks Pol II from initiating transcription from the p53RE. In fact, models of decoupled transcription factor binding and transcription initiation have been proposed recently [93]. This may be possible because the recruitment of the preinitiation complex is initiated by TFIID, which binds downstream of the TSS [94]. We speculate that TFIID may be recruited downstream of the TSS in the presence of p53 and that p53 subsequently dissociates to allow Pol II recruitment and transcription initiation. Interestingly, p53REs with synthetically high affinity for p53 binding have been shown to be poor mediators of transcription by p53 [95], suggesting that cycles of frequent association and dissociation from the DNA may be important for p53 to induce transcription. In addition, it has been shown that higher p53 levels are associated with higher target gene expression levels through increased transcription initiation frequency, i.e. burst frequency, and not through increased transcription initiation duration, i.e. burst length [96, 97]. Based on these findings and conclusions drawn from our data (Fig. 4A–D), we propose a model in which p53 increases transcription levels through increased binding dynamics, i.e. a higher frequency of on and off cycles (Fig. 4E).

## Supplementary Material

gkaf465_Supplemental_Files

## Data Availability

Recurrent p53 binding sites were taken from [41]. RFX7 binding sites were obtained from [29]. E2F4 ChIP-seq data were obtained from ENCODE (ENCFF090CEP) [98]. Enhancer–gene associations were obtained from ENCODE (ENCFF617FJH) [47]. Other sequencing data are accessible through GEO [99]. p53 ChIP-seq data were obtained from GSM2671290 and GSM2671296 [40]. CAGE-seq data are available through GSE223512 [7]. RNA-seq data are available through GSE216721 [7]. ATAC-seq data are available through GSE250017 [7]. CUT&Tag data are available through GSE278033.
