# Supplementary material for: p53 reveals principles of chromatin remodeling and enhancer activation
Source: Nucleic Acids Res. 2025 Jun 6;53(11):gkaf465. doi: 10.1093/nar/gkaf465 (PMC12143595; doi:10.1093/nar/gkaf465)
Supplement: gkaf465_Supplemental_Files [file gkaf465_supplemental_files.zip › Supplementary Information.pdf]

Supplementary Figure 1. p53 changes nucleosome occupancy. Mono-nucleosome signals inferred from DANPOS are displayed for p53 binding sites at DNA that **(A)** remained accessible/open, **(B)** became accessible upon p53 activation, and **(D)** remained closed. Regions have been sorted by DANPOS signal. Genome browser images displaying p53 ChIP-seq and ATAC-seq data at a p53 binding site that **(C)** became accessible and **(E)** remained inaccessible upon Nutlin-3a treatment. The predicted p53RE is highlighted. p53 ChIP-seq and nucleosome-free ATAC-seq data were normalized to CPM. Mono-nucleosome ATAC-seq data were normalized by DANPOS according to library size and accessible DNA background.

Supplementary Figure 2. RFX7 and E2F4 bind to promoters. (A) ATAC-seq, H3K4me1, and H3K27ac signals are displayed for RFX7 binding sites. **(B)** E2F4 ChIP-seq, ATAC-seq, H3K4me1, and H3K27ac signals are displayed for E2F4 binding sites. Regions were sorted by average signal

Supplementary Figure 3. p53 induces chromatin opening. (A) p53 ChIP-seq, ATAC-seq, H3K4me1, and H3K27ac signals are displayed for the groups of p53 binding sites identified in Fig. 1c. Regions sorted by average signal. p53 ChIP-seq, ATAC-seq, H3K4me1, and H3K27ac signals are displayed for p53 binding sites that were open in the DMSO control condition and open **(B)** or closed **(C)** in the Nutlin-3a treatment condition.

Supplementary Figure 4. Enhancer activation by p53 is similar across cell lines. Differential expression (log_2FC Nutlin-3a compared with DMSO control) of **(A)** all and **(B)** p53-bound CAGE-seq peaks compared between MCF-7, RPE-1, and U2OS cells. Spearman correlation with two-tailed significance.

Supplementary Figure 5. p53 ChIP-seq, ATAC-seq, H3K4me1, and H3K27ac signals at p53 binding sites located in **(A)** enhancers, **(B)** transcribed DNA, and **(C)** quiescent chromatin. Subgroups determined by overlaps with an induced TSS (log_2FC > 0.5), uninduced TSS (log_2FC < 0.5), and no TSS. Regions sorted by H3K27ac signal. The uninduced TSS fraction has been removed from **(A** and **C)** for visualization purposes.

Supplementary Figure 6. p53 enhancer activation correlates with associated gene expression. Differential expression measured by CAGE-seq (y-axis) displays a positive Spearman correlation with differential expression measured by RNA-seq (x-axis) for **(A)** all promoterTSS-gene connections, **(B)** all enhancerTSS-gene connections, and **(C)** p53-bound enhancerTSS-gene connections. Spearman correlation with two-tailed significance. **(D-G)** Genome browser images displaying CAGE-seq data among p53 ChIP-seq, ATAC-seq, RNA-

seq, and CUT&Tag (H3K4me1 and H3K27ac) data at p53 binding sites in enhancers linked to the p53-induced genes **(D)** *ALDH3A1*, **(E)** *HES2*, **(F)** *HMOX1*, and **(G)** *NACC2*. The predicted p53RE is highlighted. p53 ChIP-seq, nucleosome-free ATAC-seq, and CUT&Tag data were normalized to CPM. CTSSs and RNA-seq were coverage normalized. **(H)** RT-qPCR data of MCF-7 cells transfected with siTP53 or siControl and treated with Nutlin-3a or DMSO solvent control. Normalized to DMSO treatment and *ACTR10* negative control. *CDKN1A* served as a positive control for a p53-regulated gene. Mean and standard deviation is displayed; n = 3 biological replicates. Statistical significance was obtained through a two-sided unpaired t-test. *, **, and *** indicate p-values < 0.05, <0.01, and <0.001, respectively.

Supplementary Figure 7. TSS usage near p53 binding sites does not depend on chromatin remodeling. The density of CAGE-seq-derived TSSs (CTSSs) at +/- 200 bp around canonical p53REs of p53 binding sites in open (left panel) or closed (right panel) chromatin in the DMSO control condition.

Supplementary Table 1. The table contains merged ATAC-seq peaks that occur in any condition with information on differential signal assessed by DESeq2.

Supplementary Table 2. The table contains merged CAGE-seq peaks that occur in any condition with information on the differential expression assessed by DESeq2 and the position with the maximal signal, the CTSS.

Supplementary Table 3. The table contains Nutlin-3a-induced CAGE-seq peaks (log_2fold-change > 1; FDR <0.1) linked to Nutlin-3a-induced genes (log_2fold-change > 0.5; FDR < 0.05) through enhancer-gene associations from ENCODE.
