# Supplementary figures and images for: p53 reveals principles of chromatin remodeling and enhancer activation
Source: Nucleic Acids Res. 2025 Jun 6;53(11):gkaf465. doi: 10.1093/nar/gkaf465 (PMC12143595; doi:10.1093/nar/gkaf465)

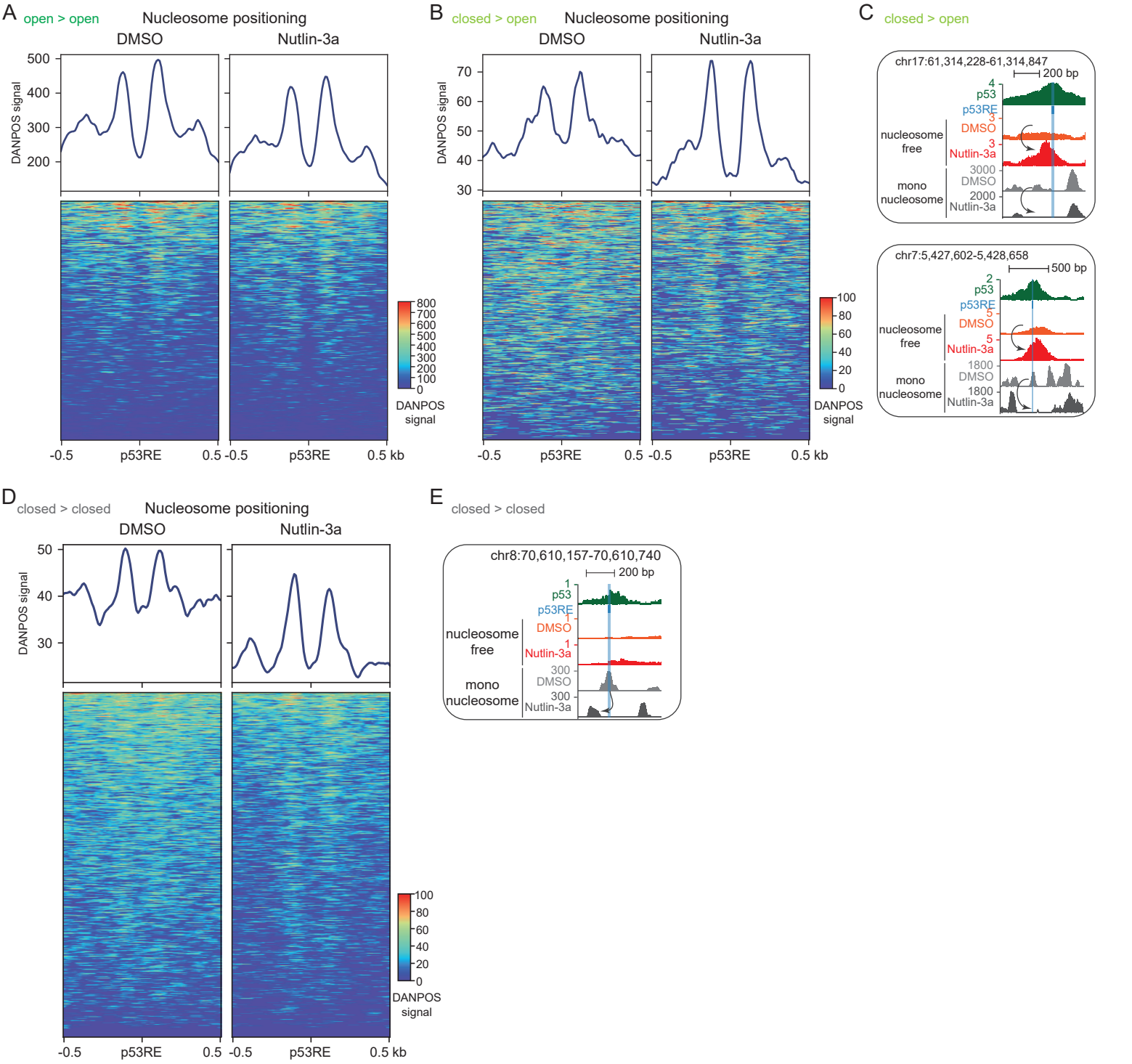

A RFX7 binding sites

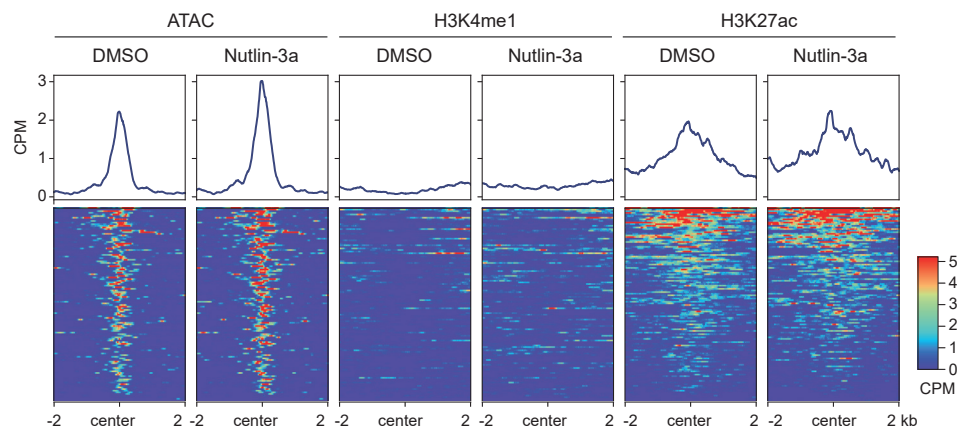

B E2F4 binding sites

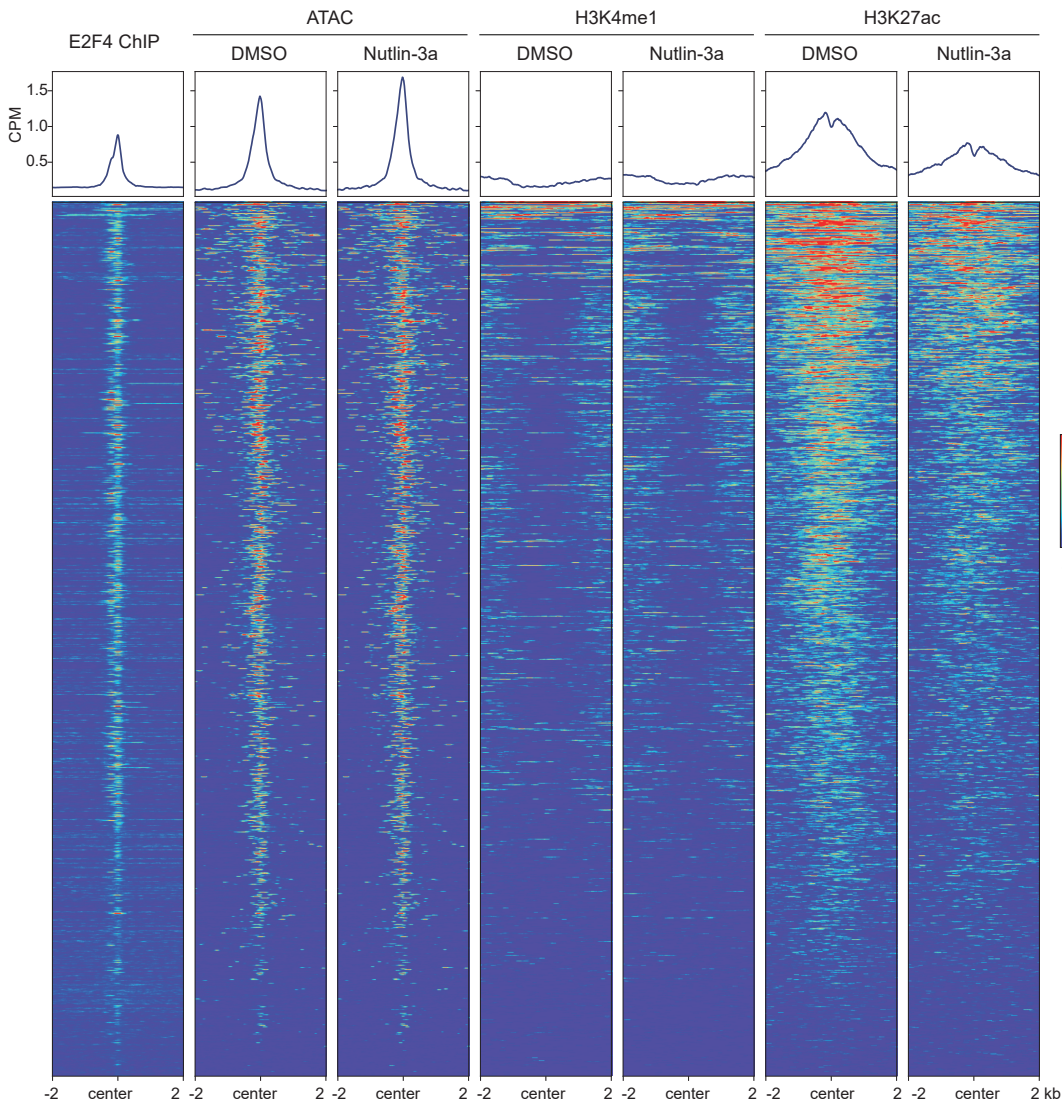

A

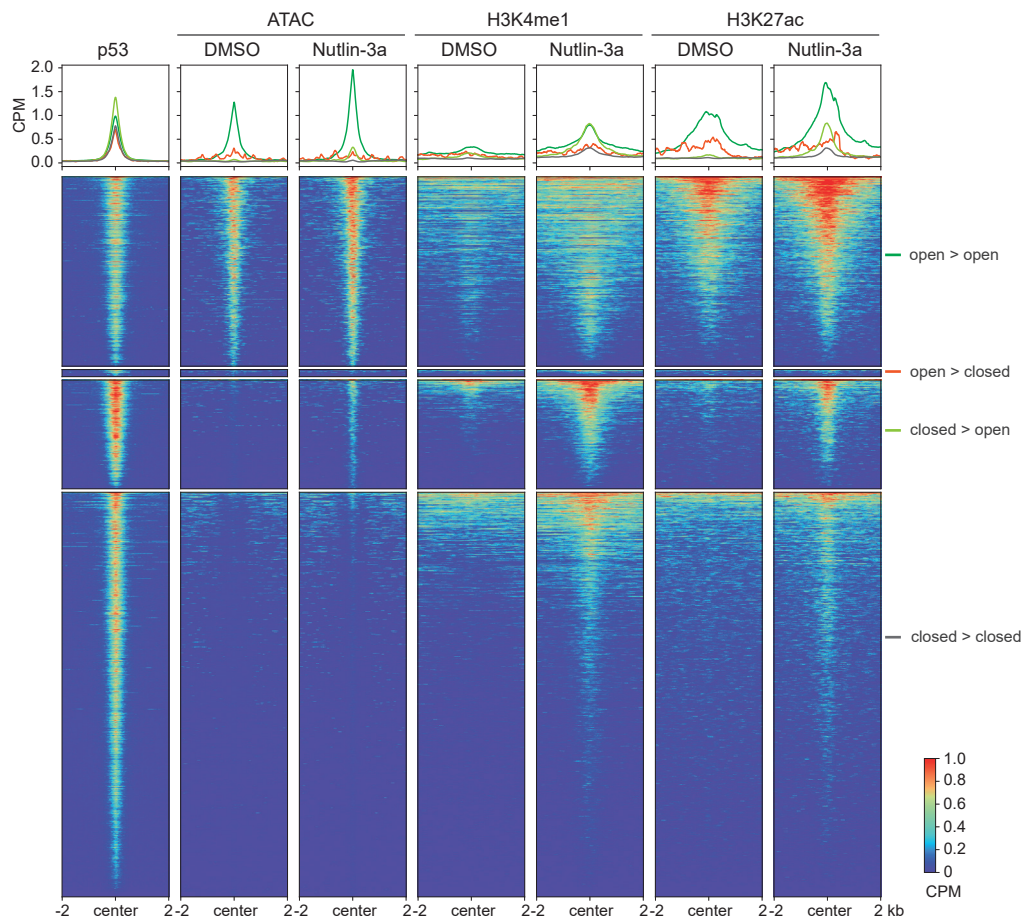

B

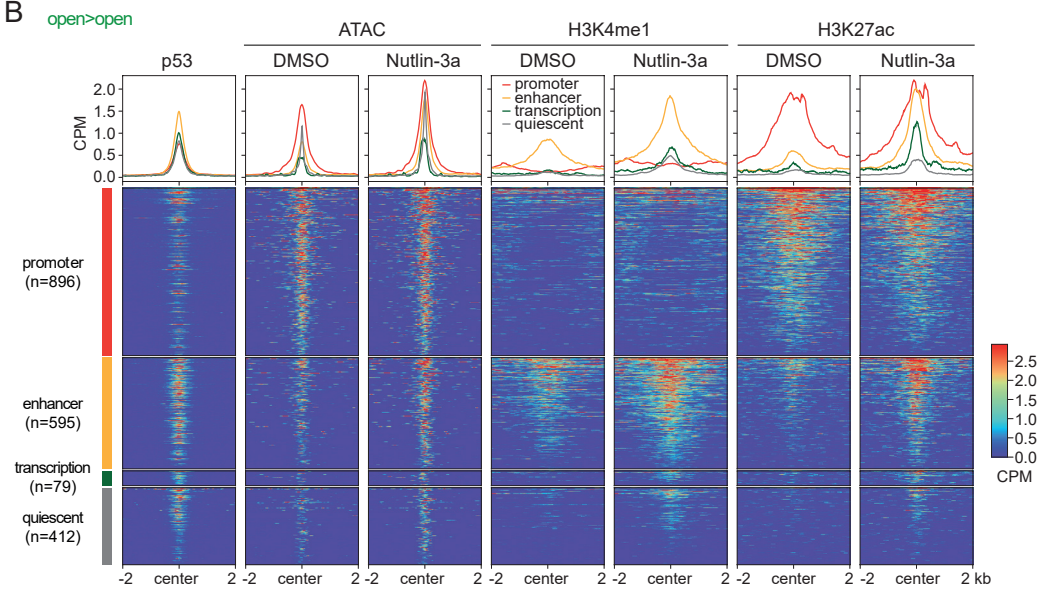

C

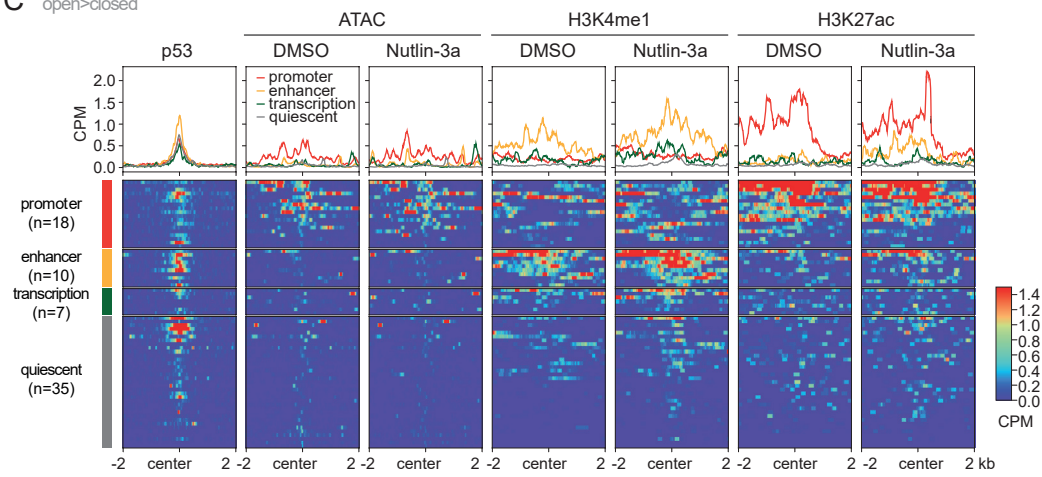

A

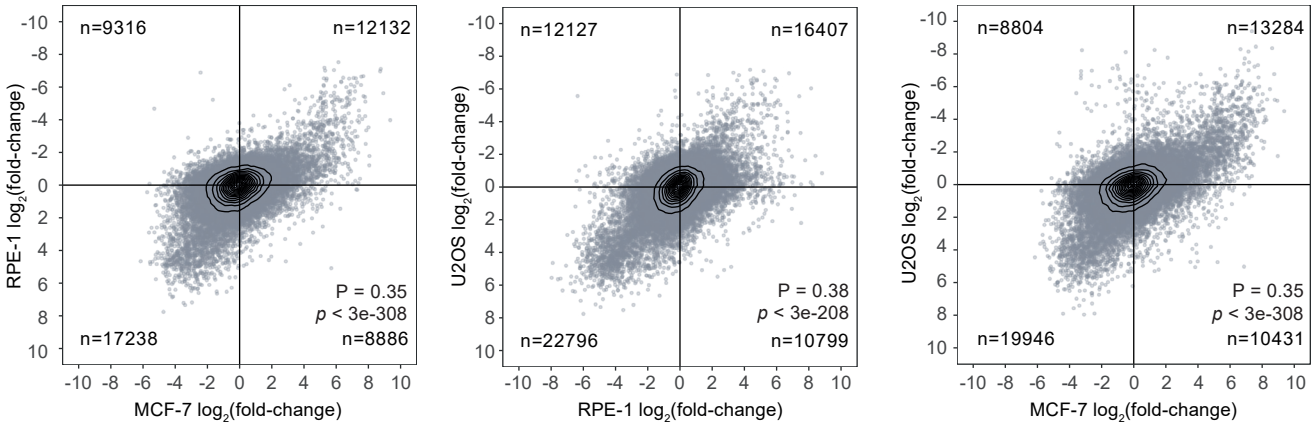

B

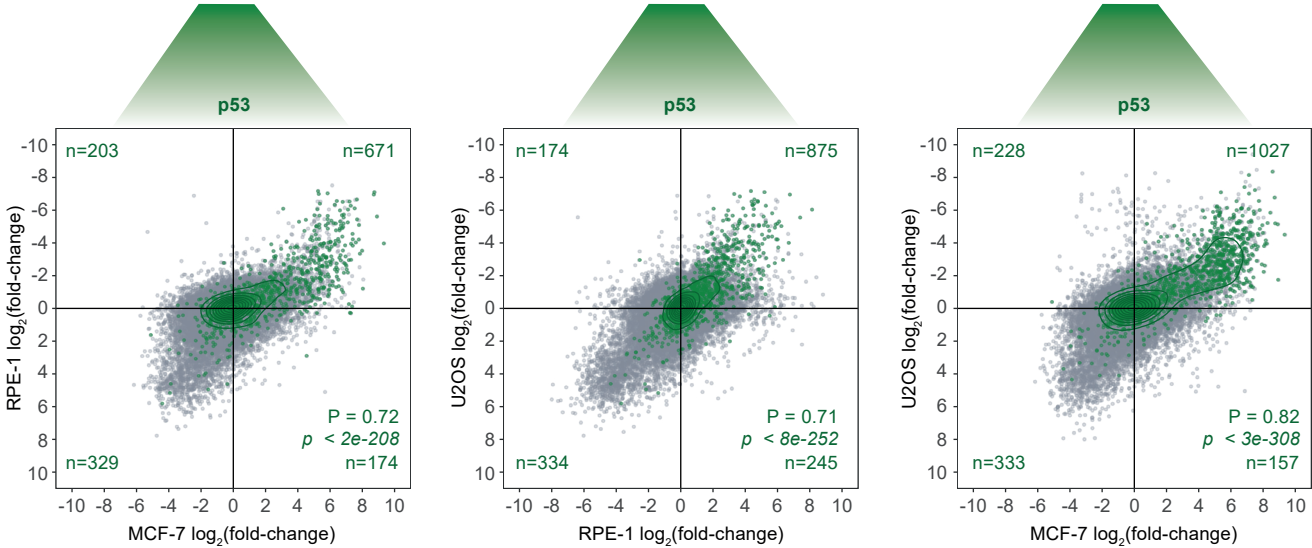

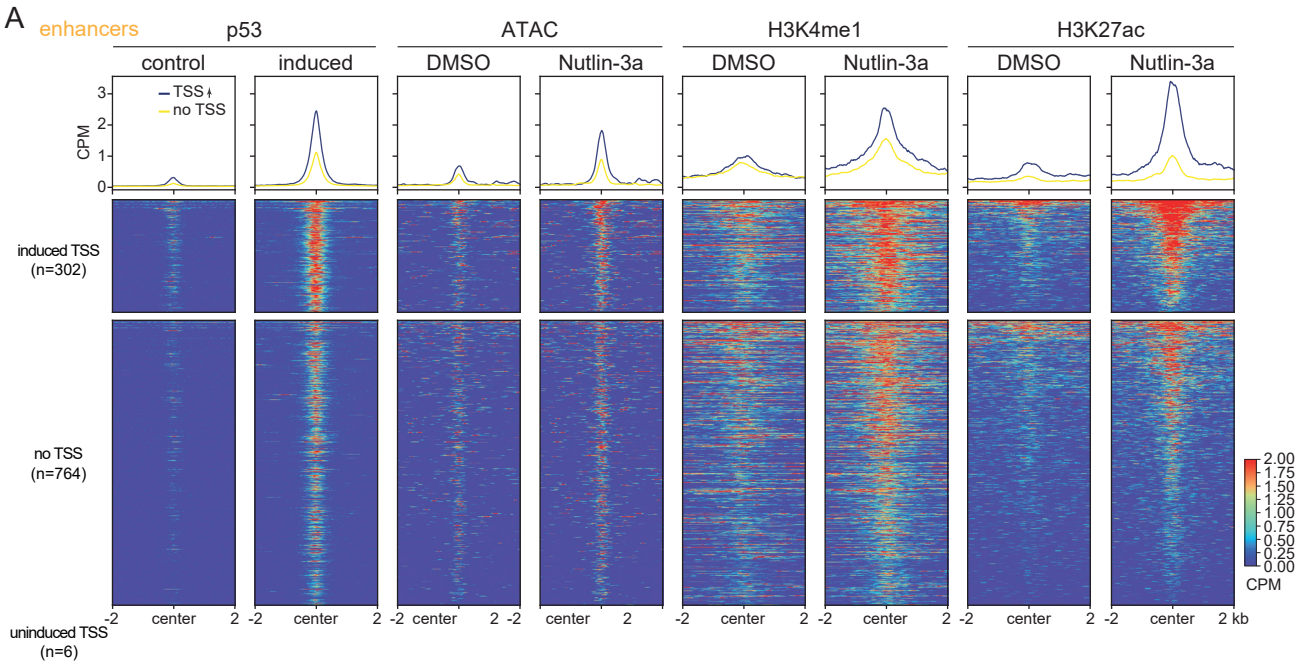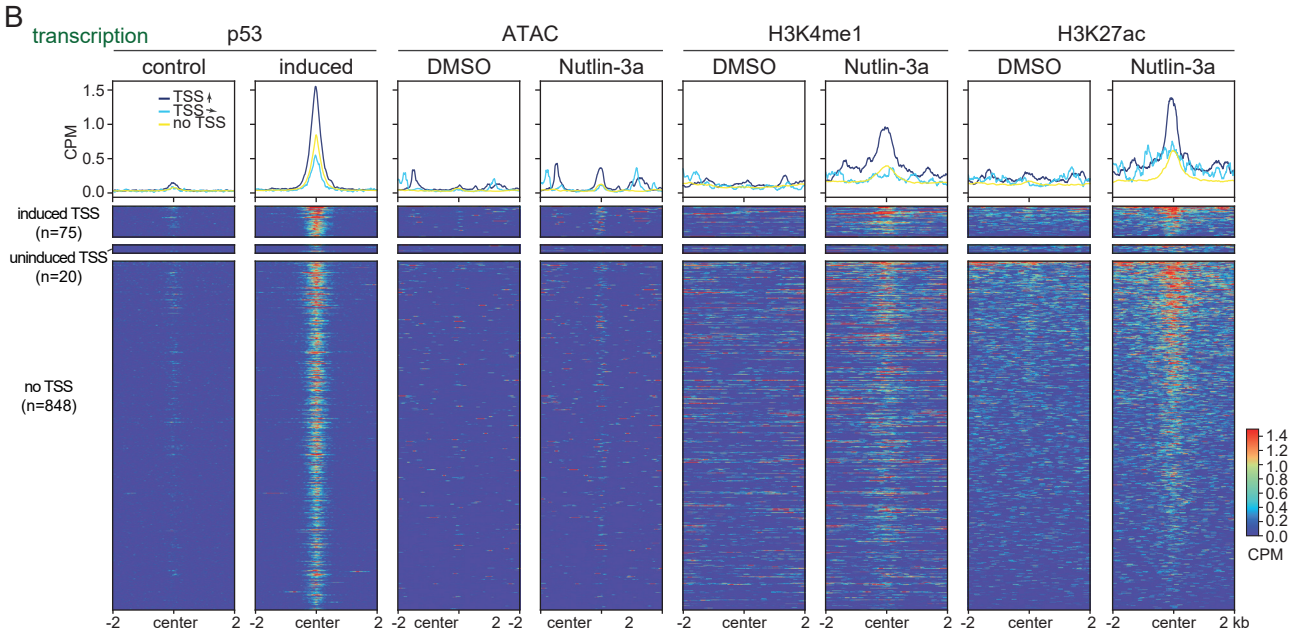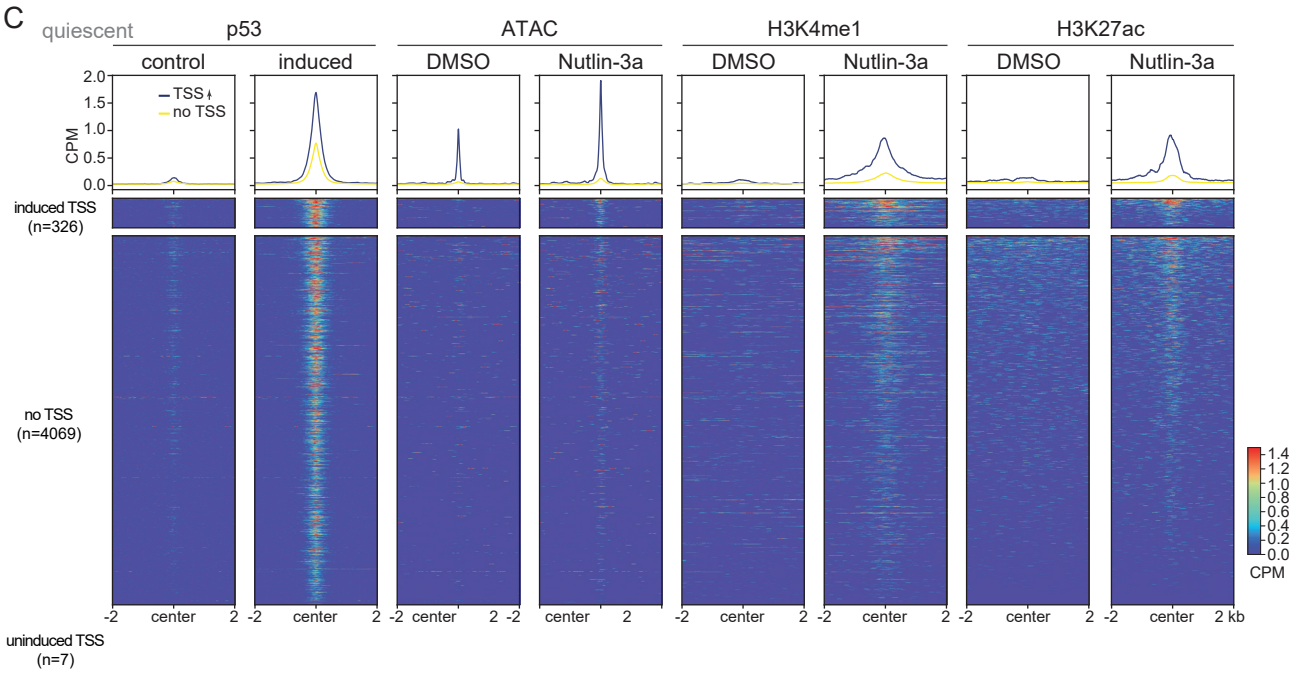

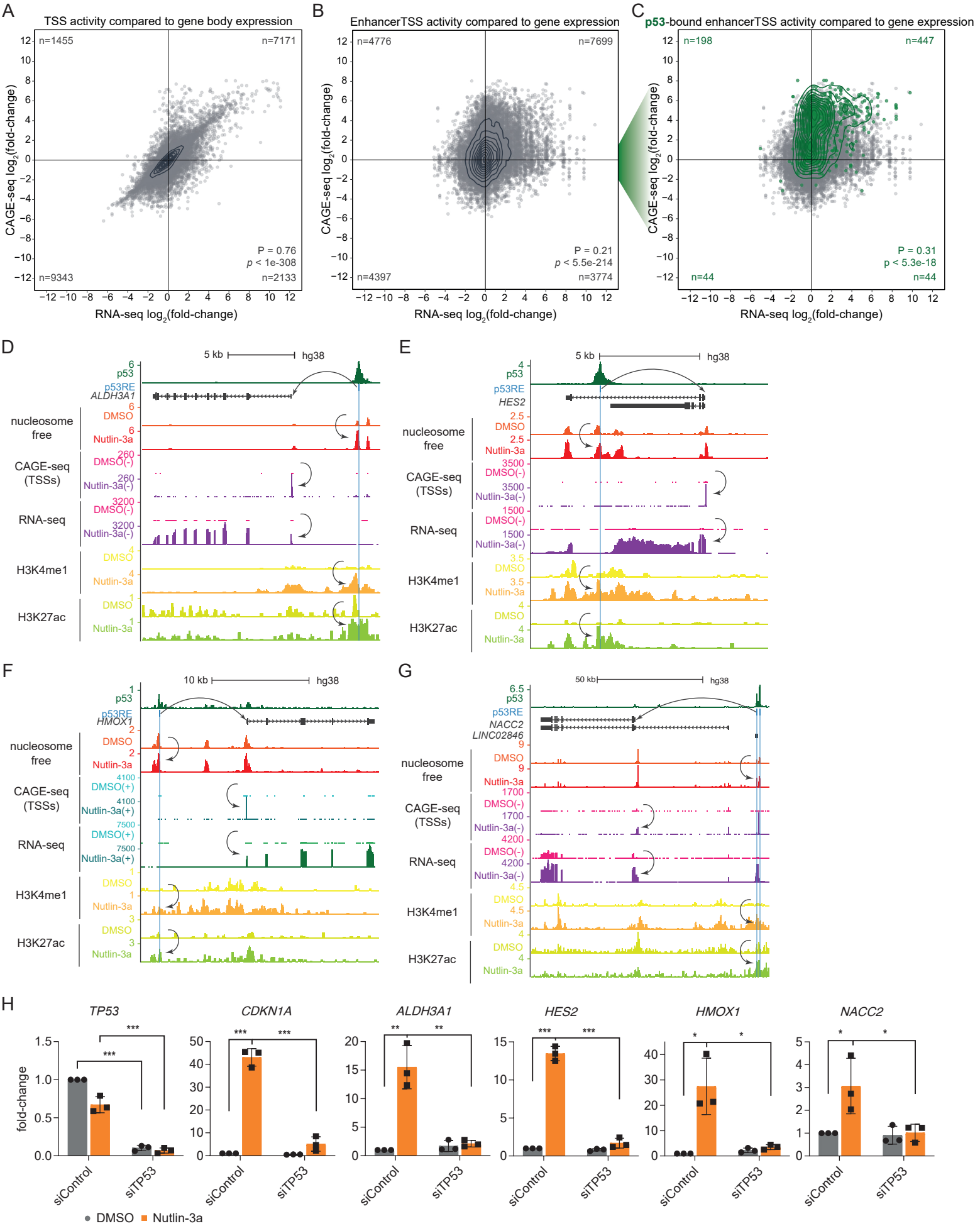

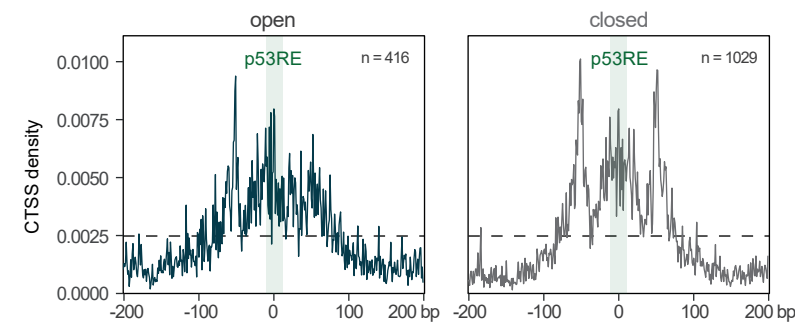

Supplement: gkaf465_Supplemental_Files [file gkaf465_supplemental_files.zip › SupplementaryFigures.pdf]
